# Comparison of the safety and efficacy of biodegradable polymer drug-eluting stents *versus* durable polymer drug-eluting stents: a meta-analysis

**DOI:** 10.1186/s40001-015-0110-z

**Published:** 2015-03-05

**Authors:** Jianfeng Lv, Yazhou Wu, Xingmei Zhang, Tao Jing, Li Zhang, Shifei Tong, Zhiyuan Song, Mingli Wang, Gang Wang, Luxiang Chi

**Affiliations:** Department of Cardiology, Southwest Hospital, Third Military Medical University, Gaotanyan Street, Shapingba District, Chongqing, 40038 China; Department of Cardiology, General Hospital of Ningxia Medical University, Shengli Street, Xingqing District, Yinchuan, 750004 China; Department of Health Statistics, Third Military Medical University, Gaotanyan Street, Shapingba District, Chongqing, 400038 China; Department of Neurology, Fifth Hospital of the People’s Liberation Army, Shengli Street, Xingqing District, Yinchuan, 750004 China

**Keywords:** Biodegradable polymer drug-eluting stent, Durable polymer drug-eluting stents, Coronary artery heart disease, Meta-analysis

## Abstract

**Background:**

A meta-analysis was conducted to assess the safety and efficacy of biodegradable polymer drug-eluting stents (BP-DESs).

**Methods:**

PubMed, Science Direct, China National Knowledge Infrastructure, and Chongqing VIP databases were searched for randomized controlled trials comparing the safety and efficacy of BP-DESs *versus* durable polymer drug-eluting stents (DP-DESs). Efficacy included the prevalence of target lesion revascularization (TLR), target vessel revascularization (TVR), and late lumen loss (LLL), and safety of these stents at the end of follow-up for the selected research studies were compared.

**Results:**

A total of 16 qualified original studies that addressed a total of 22,211 patients were included in this meta-analysis. In regard to efficacy, no statistically significant difference in TLR (odds ratio (OR) = 0.94, *P* = 0.30) or TVR (OR 1.01, *P* = 0.86) was observed between patients treated with BP-DESs and those with DP-DESs. However, there were significant differences in in-stent LLL (weighted mean difference [WMD] = −0.07, *P* = 0.005) and in-segment LLL (WMD = −0.03, *P* = 0.05) between patients treated with BP-DESs and with DP-DESs. In terms of safety, there was no significant difference in overall mortality (OR 0.97, *P* = 0.67), cardiac death (OR 0.99, *P* = 0.90), early stent thrombosis (ST) and late ST (OR 0.94, *P* = 0.76; OR 0.96, *P* = 0.73), or myocardial infarction (MI) (OR 0.99, *P* = 0.88) between patients treated with BP-DESs and with DP-DESs. However, there was a statistically significant difference in very late ST (OR 0.69, *P* = 0.007) between these two groups. In addition, the general trend of the rates of TVR and TLR of BP-DESs groups was lower than DP-DESs groups after a 1-year follow-up.

**Conclusion:**

BP-DESs are safe, efficient, and exhibit superior performance to DP-DESs with respect to reducing the occurrence of very late ST and LLL. The general trend of the rates of TVR and TLR of BP-DESs groups was lower than DP-DESs groups after a 1-year follow-up.

## Background

Recent discoveries in medical technology and bioengineering have led to substantial advancements in the design of coronary stents, particularly in lowering the rates of restenosis occurrence after percutaneous coronary stenting with drug-eluting stents (DESs) rather than after percutaneous coronary stenting with bare metal stents (BMSs) [1]. A large number of studies have demonstrated that the presence of a non-degradable polymer in the coronary arteries after prolonged drug release can cause inflammatory responses in local vessel walls and delay the healing of the vascular endothelium [2-4]. These issues may be the primary causes of postoperative stent thrombosis and late stenosis following the implantation of permanent durable polymer drug-eluting stents (DP-DESs) [5,6]. It has been proven that the implantation of a first-generation DP-DES instead of a BMS can decrease both the incidence of restenosis and target lesion revascularization after percutaneous coronary intervention (PCI) [7]. The underlying mechanism leading to restenosis is an excessive local vascular healing response to balloon and stent injury; this response stimulates the release of a variety of growth factors and cytokines, causing vascular smooth muscle cell and intimal proliferation and thereby resulting in the thickening of blood vessel walls [8]. A coating of antiproliferative drugs and polymers on the stent will not only inhibit the proliferation and migration of smooth muscle cells but also affect the function of endothelial cells. This effect can result in the poor healing of stent-injured vascular segments; moreover, the long-term presence of a permanent polymer stent coating can produce a persistent inflammatory response. This inflammation can cause the proliferation of endothelial cells in stented vessel segments, resulting in restenosis. The recent invention of BP-DESs can solve this issue; in particular, the complete degradation of the polymer coating of BP-DESs will ensure that BP-DESs cause significantly lower levels of long-term structural damage and chronic inflammation than DP-DESs that feature a permanent polymer coating.

A new generation of BP-DESs has been developed that exhibit good potential applicability because they not only effectively deliver drugs to patients but will also completely degrade into harmless compounds such as water, and carbon dioxide after drug release has been completed [9].

Relative to the use of BMSs, the use of DESs and polymer-free DESs can produce significantly lower restenosis rates after PCI; thus, the development of DESs constitutes a milestone in the field of interventional cardiology. Although the use of DESs instead of BMSs can significantly improve the therapeutic efficacy of PCI treatments, previous studies have reported that compared to implantation of BMSs, the implantation of DESs produces significantly higher incidences of adverse events, such as stent thrombosis (ST) [10]. The incidence of ST is relatively low; nonetheless, this issue has attracted widespread concern because the consequences of ST are extremely serious [11,12]. Moreover, investigations have indicated that the presence of a DES may delay vascular endothelial healing and that the permanent residues left by non-degradable polymers from such stents may trigger an inflammatory response in local vessel walls; these phenomena are closely related to severe adverse reactions to DESs [13,14]. Over time, the drug-coated polymers of BP-DESs can gradually degrade into completely harmless CO_2_ and H_2_O molecules that are excreted from a patient’s body. In this manner, a BP-DES can completely transform into a BMS following a slow, controlled drug release. Thus, relative to DP-DESs, BP-DESs can reduce drug-induced delays in vascular endothelialization and inflammatory responses of local vessel walls caused by the presence of a permanent polymer, thereby achieving the dual purpose of preventing both in-stent restenosis and late stent thrombosis [10].

Several previous studies regarding the efficacy and safety of BP-DESs and DP-DESs have produced inconsistent results [15-17]. However, as mentioned in another paper, the major limitations of the previous meta-analyses were that the variability of patients with newer-generation DES were relatively small and thus comparisons had restricted statistical power, and BP-DESs were not included in the analyses. Even though, BP-DES is not approved in the USA, they are still used worldwide including some countries in Asia and Europe. Therefore, we conducted this meta-analysis to compare the safety and efficacy of these two types of stents, with the inclusion of Chinese research studies on this topic.

## Methods

### Study design

A systematic literature search was performed to identify randomized controlled studies (RCTs) assessing safety and efficacy of BP-DESs and DP-DESs in accordance with the recommended steps provided in the Cochrane Handbook for Systematic Reviews of Interventions [18]. Results were systematically analyzed to determine the relationship between treatment methods and its efficacy and safety.

### Database search

Electronic searches were performed using the electronic databases provided by PubMed, Science Direct, China National Knowledge Infrastructure, Chongqing VIP, and other databases for relevant studies published between December 1990 and December 2015. The literature searches were conducted using the search terms “biodegradable polymer,” “drug-eluting,” “stent,” and “coronary disease” with various combinations of the operators “AND,” “NOT,” and “OR”. The articles written in English and/or Chinese language were included.

### Study inclusion and exclusion criteria

The studies examined in this study were required to fulfill the following inclusion criteria: (1) human study subjects, (2) a randomized controlled design, relatively complete study results, (4) a follow-up period of more than 6 months, and (5) an experimental comparison between BP-DESs and DP-DESs with respect to safety and efficacy. The following exclusion criteria were utilized for the examined studies: (1) a non-randomized controlled design, (2) incomplete data, the use of BMSs in a control group, and (4) a repeated examination of the same study population (in these cases, only the study with the longest follow-up period was utilized). Studies were selected in strict accordance with the inclusion and exclusion criteria. All of the original reports for the selected studies included specific diagnostic criteria, inclusion criteria for the examined cases, appropriate statistical methods, explicit evaluation indicators, and clear and complete data.

### Data collection and outcome measurement

Two authors (Gang Wang and Mingli Wang) independently assessed all potentially relevant studies and reached a consensus on all items. Any discrepancies were resolved through discussion, with arbitration by a third author if necessary. The following data were collected from each study: first author, publication year, characteristics of study subjects, study design, outcomes for efficacy, and safety of stents (Tables 1 and 2).

**Table 1:** **The quality outcomes of assessed clinical studies that were included**

**Author**	**Randomized approach**	**Allocation concealment**	**Blinding**	**Loss to follow-up**	**Jadad score**	**Clinical outcomes**
Chevalier [19]	2	2	1	0	5	Cardiac death, overall mortality, MI, TLR, TVR, ST, LLL
Byrne [20]	2	2	2	1	7	Overall mortality, MI, TLR, ST, LLL
Chevalier [21]	2	2	1	1	6	Cardiac death, overall mortality, MI, TLR, TVR, ST, LLL
Stefanini [22]	2	2	2	1	7	Cardiac death, overall mortality, MI, TLR, TVR, ST
Byrne [23]	2	2	2	1	7	Cardiac death, overall mortality, MI, TLR, ST
Maamoun [24]	1	1	0	1	3	Cardiac death, overall mortality, MI, TLR, ST
Kadota [25]	1	1	0	1	3	Cardiac death, overall mortality, MI, TLR, TVR, ST, LLL
Xu [26]	1	1	2	0	4	Cardiac death, overall mortality, MI, TLR, ST, LLL
Tan [27]	1	1	2	1	5	Cardiac death, overall mortality, MI, TLR, TVR, ST
Ge [28]	1	1	0	1	3	Cardiac death, MI, TVR, ST
Smits [29]	2	2	0	0	4	Cardiac death, overall mortality, MI, TVR, TLR, ST
Zhang [30]	2	2	1	1	6	Cardiac death, overall mortality, MI, TVR, TLR, ST
Natsuaki [31]	2	2	0	0	4	Cardiac death, overall mortality, MI, TVR, TLR, ST
Raungaard [32]	2	2	0	0	4	Cardiac death, overall mortality, MI, TVR, TLR, ST
Han [33]	2	1	1	1	5	Cardiac death, overall mortality, MI, TVR, TLR, ST
Pilgrim [34]	2	2	2	0	6	Cardiac death, overall mortality, MI, TVR, TLR, ST

*MI*, myocardial infarction; *TLR*, target lesion revascularization; *TVR*, target vessel revascularization; *ST*, stent thrombosis; *LLL*, late lumen loss.

**Table 2:** **The baseline characteristics of the included studies**

**Author**	**Source**	**Type**		**NC**	**DAPT (months)**	**FU (months)**	**Age (BP/DP)**	**Male (BP/DP)**	**Diabetes (BP/DP) (%)**	**Type B2/C (BP/DP) (%)**	**Lesion length (mean ± SD)**		**Reference diameter (mean ± SD)**	
		**BP**	**DP**								**BP**	**DP**	**BP**	**DP**
Chevalier [19]	EuroInterv 2007	BES	PES	120	6	9	65/63	69/66	18/26	56.47/68.57	11.35 ± 4.51	11.03 ± 4.75	2.70 ± 0.44	2.60 ± 0.57
Byrne [20]	Heart 2009	SES	SES	404	12	24	66.5/65	79.3/78.2	27.4/28.7	NA	13.9 ± 7.2	14.6 ± 7.0	2.74 ± 0.59	2.80 ± 0.52
Chevalier [21]	Circ Cardiovasc Intervent 2009	BES	PES	243	6	9	62.7/63.2	74.5/68.9	16.3/27.8	49.67/44.44	10.56	10.84	2.72	2.73
Stefanini [22]	Lancet 2011	BES	SES	1,707	12	48	64.6/64.5	75.0/74.6	26.0/22.5	NA	15.2 ± 11.7	14.4 ± 10.6	2.60 ± 0.61	2.71 ± 0.52
Byrne [23]	JACC 2011	SES	EESSES	2,603	6	36	66.7/66.8	77.8/75.9	28.2/29.6	72.8/72.1	14.8 ± 8.6	15.0 ± 8.8	2.79 ± 0.47	2.75 ± 0.51
Maamoun [24]	The Egyptian Heart Journal 2012	BES	SESPES	145	6	24	56.7/54.2	87.1/85.5	NA	NA	NA	NA	NA	NA
Kadota [25]	CCI 2012	BES	SES	326	3	9	67.1/67.7	71.6/72.0	38.7/39.4	69.7/62.0	12.64 ± 5.52	12.82 ± 6.81	2.68 ± 0.57	2.68 ± 0.54
Xu [26]	EuroInterv 2012	SES	SES	300	12	24	56.6/56.7	66.7/72	22/20	47.0/41.7	17.74 ± 9.30	18.72 ± 9.98	NA	NA
Tan [27]	Chin J Clinicians 2012	BES	EES	50	12	24	62/63	70.0/73.3	20.0/23.3	NA	NA	NA	NA	NA
Ge [28]	JACC 2012	SES	SES	1,909	12	12	63.3/62.1	67.7/73.0	20.5/20.75	NA	NA	NA	NA	NA
Smits [29]	Lancet 2013	BES	EES	2,707	12	12	63/62.7	74.4/74.3	21.8/21.6	63.7/63.3	16.8 ± 9.8	17.7 ± 10.6	2.9 ± 0.5	2.9 ± 0.5
Zhang [30]	INT J CARDIOL 2013	SES	SES	662	12	24	65.9/67.5	68.5/69.2	27.73/32.26	66.34/69.51	24.77 ± 14.47	29.20 ± 16.6	3.13 ± 0.45	3.26 ± 2.40
Natsuaki [31]	JACC 2013	BES	EES	3,235	3	12	69.1/69.3	77/77	46/46	NA	19.5 ± 12.8	19.3 ± 13.1	2.62 ± 0.6	2.61 ± 0.57
Raungaard [32]	Lancet 2015	BES	ZES	2,999	12	12	65.8/65.7	76.2/75.8	17.6/18.0	63/70	15.0	16.0	3.0	3.2
Han [33]	JACC 2014	SES	SES	2,737	6	12	60.2/60.2	68/70	22.6/21.3	83.5/85.1	20.6 ± 12.3	21.2 ± 12.9	2.79 ± 0.47	2.79 ± 0.44
Pilgrim [34]	Lancet 2014	SES	EES	2,119	12	12	66.1/65.9	77.0/77.3	24.2/21.7	NA	25.91 ± 15.40	27.45 ± 16.77	3.05 ± 0.49	3.03 ± 0.49

Notes: *BP*, bioabsorbable polymer drug-eluting stent; *DP*, durable polymer drug-eluting stent; *NC*, number of cases; *DAPT*, dual antiplatelet therapy; *FU*, follow-up period; *BES*, biolimus-eluting stent; *SES*, sirolimus-eluting stent; *PES*, paclitaxel-eluting stent; *EES*, everolimus-eluting stent; *ZES*, zotarolimus-eluting stent; *NA*, not available. *Mean ± SD*, the mean lesion length ± the standard deviation of this length.

The included studies assessed indicators of the efficacy and safety of stents. The efficacy indicators included the prevalence of target lesion revascularization (TLR), target vessel revascularization (TVR), and late lumen loss (LLL) (including both in-segment and in-stent LLL) during the follow-up period. The safety indicators included not only overall mortality and cardiac deaths but also the prevalence of myocardial infarction (MI) and stent thrombosis (ST) during follow-up periods.

### Quality assessment

Eligible studies were evaluated for inclusion by two independent reviewers (Xingmei Zhang and Li Zhang), and the level of agreement between reviewers was recorded. Inclusion of studies was determined by screening of manual titles and abstracts, followed by full-text screening by the same reviewers. The quality of each study was assessed using the modified Jaded scale for study selection [35] (for more detailed information, see Table 1). These scales were used to evaluate randomized approach, allocation concealment, blinding, and lost to follow-up. In the event of incomplete data, authors of potentially eligible studies were contacted to obtain relevant unpublished data.

### Statistical analysis

Review Manager (RevMan) 5.1 statistical software package (Cochrane Collaboration, Copenhagen, Denmark) was utilized to perform this meta-analysis. The odds ratio (OR) and weighted mean difference (WMD) statistics were used for the analysis of efficacy-related count data and measurement data, respectively. Additionally, 95% confidence intervals (CIs) were provided for all efficacy analyses, and forest plot was created. A funnel plot of the included studies was created to detect the presence of publication bias. The heterogeneity of the results of clinical trials was assessed by the chi-square test. If these results were homogeneous (*P* > 0.1, *I*^2^ < 50%), fixed effects models were utilized for meta-analyses, whereas if these results were heterogeneous (*P* ≤ 0.1, *I*^2^ ≥ 50%), the causes of this heterogeneity were assessed and a sensitivity analysis was performed. If heterogeneities in the results of clinical trials could not be eliminated, random effects models were used for meta-analyses or qualitative systemic evaluations were conducted. A *P* value of ≤0.05 was regarded as statistically significant.

## Results and discussion

### Literature search

**Figure 1:**
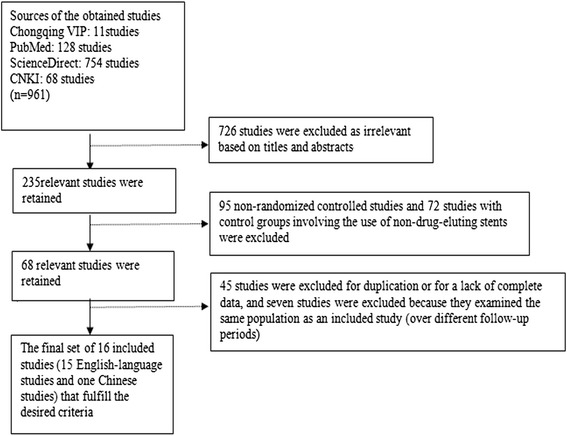
**A flowchart of the literature review process.**

A total of 961 original studies, including 882 English-language publications and 79 Chinese publications, were retrieved from internet searches of electronic databases. However, 726 of these studies were excluded as irrelevant based on their titles and abstracts, 95 of these studies were excluded because they were non-(RCTs), and an additional 72 of these studies were excluded because their control groups involved the utilization of non-drug-eluting stents. Thus, 68 original studies were initially obtained. After 45 of these studies were excluded due to either duplication or a lack of complete data, a total of 23 studies were regarded as relevant investigations. Seven of these studies were excluded because they involved examinations of the same population over different follow-up periods. Eventually, 16 studies were identified that fulfilled the study selection criteria (including 15 English-language publications and 1 Chinese publication) (Figure 1).

### Characteristics of included studies

All of these included studies were randomized controlled investigations that specified the number of studied cases and the number of occurrences of MI. Out of the 16 chosen literature, 15 studies provided clear data regarding overall mortality and clear data regarding the occurrence of ST and cardiac death. Fourteen studies provided clear data regarding the occurrence of TLR, 12 studies provided clear data regarding the occurrence of TVR, and 6 studies provided clear data regarding the occurrence of LLL (Table 1). The selected literature included one conference abstract; however, detailed data regarding the study described by this abstract were obtained in PowerPoint (ppt) format. Full-text reports describing all of the remaining included studies have been published. For all of the included studies, there were no significant differences in the baseline characteristics of the cases in the BP-DES and the DP-DES groups (Table 2). The follow-up periods of the studies examined in this investigation ranged from 6 to 48 months. The selected efficacy indicators included TLR, TVR, and LLL (including both in-segment and in-stent LLL); the selected safety indicators included overall mortality, cardiac deaths, MI, and ST.

### Efficacy analysis

**Figure 2:**
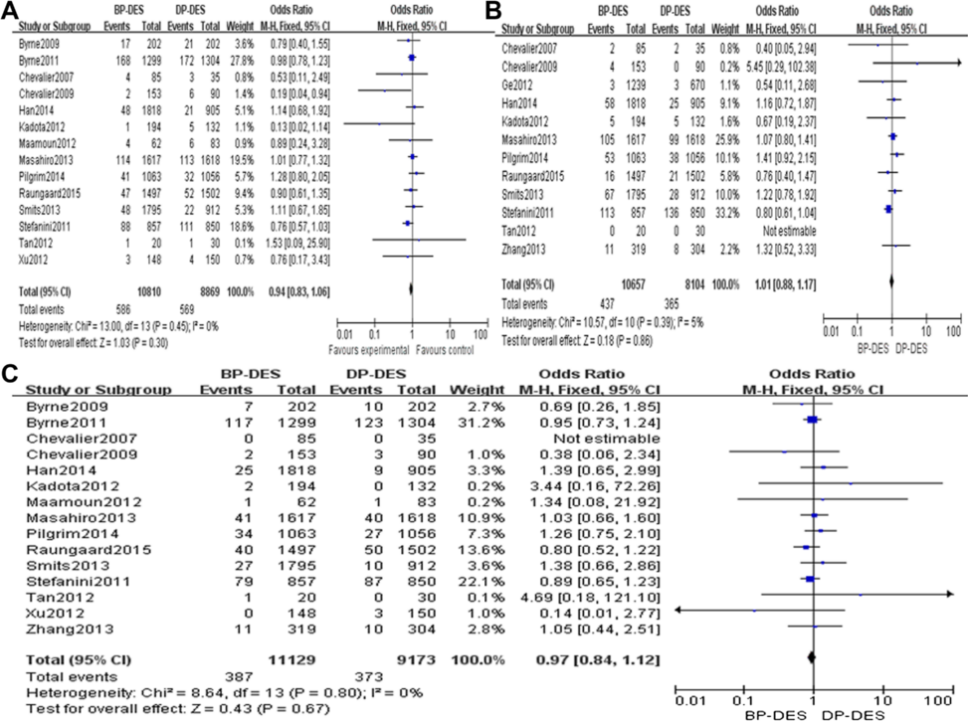
**Fixed effects model used for the TLR-related meta-analysis. (A)** The TLR forest plot for patients with coronary artery heart disease who received a BP-DES or a DP-DES. **(B)** The TVR forest plot for patients with coronary artery heart disease who received a BP-DES or a DP-DES. **(C)** The total mortality forest plot for patients with coronary artery heart disease who received a BP-DES or a DP-DES.

**Figure 3:**
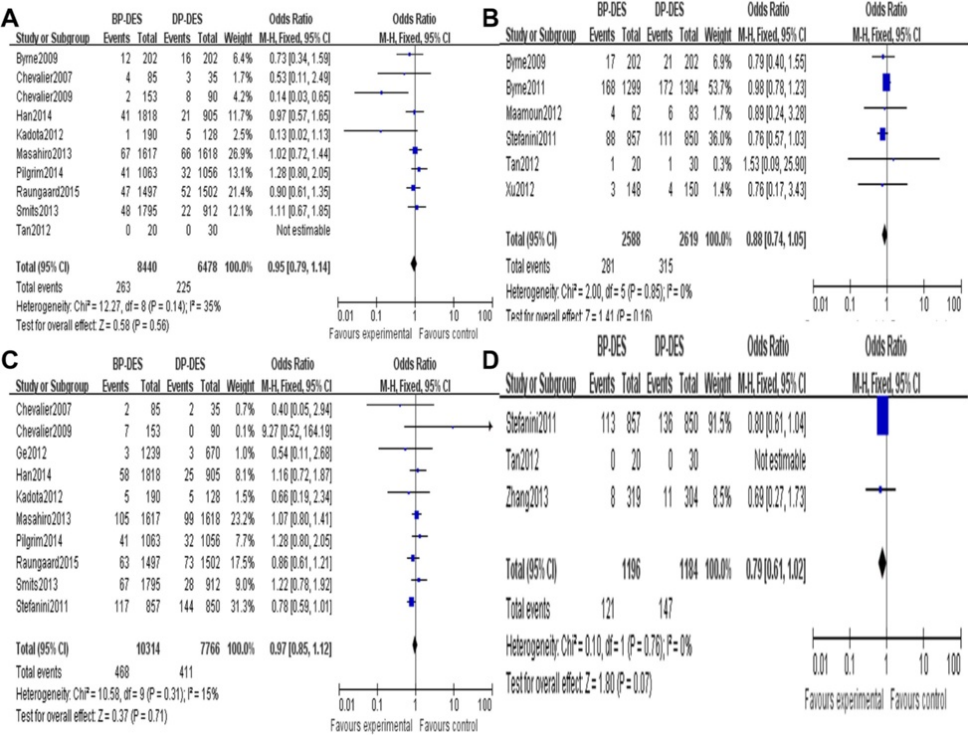
**General trend of the rates of TVR and TLR of DP-DES. (A)** The TLR forest plot for patients with coronary artery heart disease who received a BP-DES or a DP-DES during 1 year. **(B)** The TLR forest plot for patients with coronary artery heart disease who received a BP-DES or a DP-DES more than 1 year. **(C)** The TVR forest plot for patients with coronary artery heart disease who received a BP-DES or a DP-DES during 1 year. **(D)** The TVR forest plot for patients with coronary artery heart disease who received a BP-DES or a DP-DES more than 1 year.

There was no significant heterogeneity regarding the occurrence of TLR and different times TLR among the 14 included studies that addressed this phenomenon (*P* = 0.45, *I*^2^ = 0%; *P* = 0.14, *I*^2^ = 35%; *P* = 0.85, *I*^2^ = 0%); therefore, a fixed effects model was used for the TLR-related meta-analysis. There was no significant difference between the BP-DES and DP-DES groups with respect to the occurrence of TLR during follow-up periods (OR 0.94, 95% CI 0.83 to 1.06, *Z* = 1.03, *P* = 0.30, Figure 2A). Also there was no significant difference between the BP-DES and DP-DES groups with respect to the occurrence of TLR during 1-year and more than 1-year follow-up periods, but we found the general trend of the rates of TLR of DP-DES groups was significantly higher (OR 0.95, 95% CI 0.79 to 1.14, *Z* = 0.58, *P* = 0.56; OR 0.88, 95% CI 0.74 to 1.05, *Z* = 1.41, *P* = 0.16, Figure 3A, B).

There was no significant heterogeneity regarding the occurrence of TVR and different times TVR among the 12 included studies that addressed this phenomenon (*P* = 0.39, *I*^2^ = 5%; *P* = 0.58, *I*^2^ = 0%; *P* = 0.76, *I*^2^ = 0%); therefore, a fixed effects model was used for the TVR-related meta-analysis. There was no significant difference between the BP-DES and the DP-DES groups with respect to the occurrence of TVR during follow-up periods (OR 1.01, 95% CI 0.88 to 1.17, *Z* = 0.18, *P* = 0.86, Figure 2B). Also there was no significant difference between the BP-DES and DP-DES groups with respect to the occurrence of TVR during 1-year and more than 1-year follow-up periods, but we found that the general trend of the rates of TVR of DP-DES was significantly higher (OR 1.00, 95% CI 0.82 to 1.21, *Z* = 0.01, *P* = 0.99; OR 0.79, 95% CI 0.61 to 1.02, *Z* = 1.80, *P* = 0.07, Figure 3C, D).

**Figure 4:**
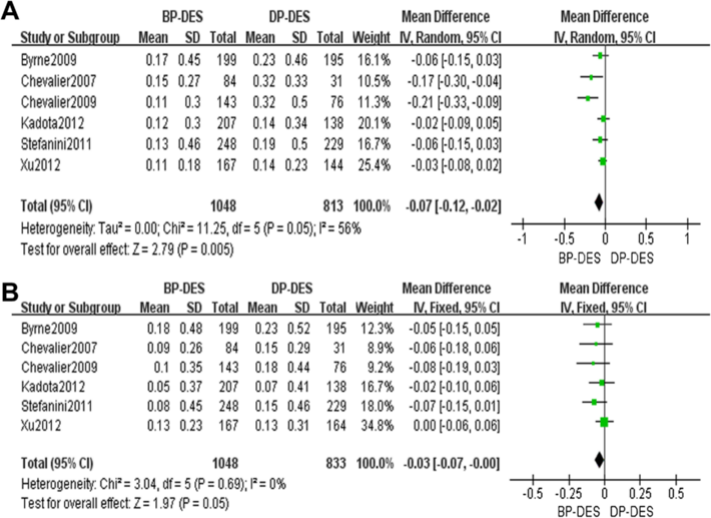
**Details regarding the LLL-related meta-analyses. (A)** The in-stent LLL forest plot for patients with coronary artery heart disease who received a BP-DES or a DP-DES. **(B)** The in-segment LLL forest plot for patients with coronary artery heart disease who received a BP-DES or a DP-DES.

There was a significant heterogeneity regarding in-stent LLL among the six included studies that addressed this phenomenon (*P* = 0.05, *I*^2^ = 56%); therefore, a random effects model was used for the meta-analysis of in-stent LLL. This meta-analysis revealed a significant difference between the BP-DES and DP-DES groups with respect to in-stent LLL during follow-up periods (WMD = −0.07, 95% CI −0.12 to −0.02, *Z* = 2.79, *P* = 0.005), suggesting that in-stent LLL was significantly lower in the BP-DES group than in the DP-DES group. The six included studies were examined to assess in-segment LLL. There was no significant heterogeneity among these studies with respect to in-segment LLL (*P* = 0.69 > 0.1, *I*^2^ = 0%); therefore, a fixed effects model was used. In-segment LLL was significantly lower (WMD = −0.03, 95% CI −0.07 to −0.00, *Z* = 1.97, *P* = 0.05) in the BP-DES group than in the DP-DES group. One set of relevant studies that reported LLL data conducted repeated measurements of the same population; the report from this set of studies that involved the longest follow-up period was selected as the included study for this investigation. However, for the LLL analyses, the relevant data for this population were instead obtained from a study with a short follow-up period because the other LLL data of this investigation were obtained from studies with relatively short follow-up periods [36]. Details regarding the LLL-related meta-analyses are provided in Figure 4A, B).

### Safety analysis

**Figure 5:**
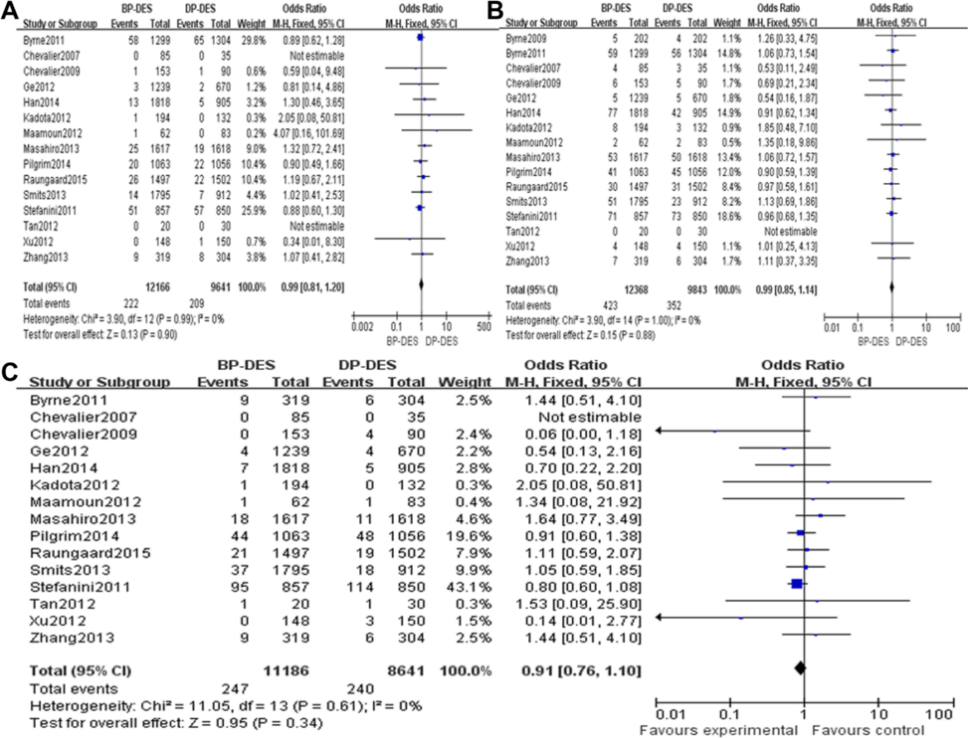
**Results of the BP-DES and DP-DES groups with respect to overall mortality. (A)** The cardiac death forest plot for patients with coronary artery heart disease who received a BP-DES or a DP-DES. **(B)** The MI forest plot for patients with coronary artery heart disease who received a BP-DES or a DP-DES. **(C)** A stent thrombosis (ST) forest plot for patients with coronary artery heart disease who received a BP-DES or a DP-DES.

**Figure 6:**
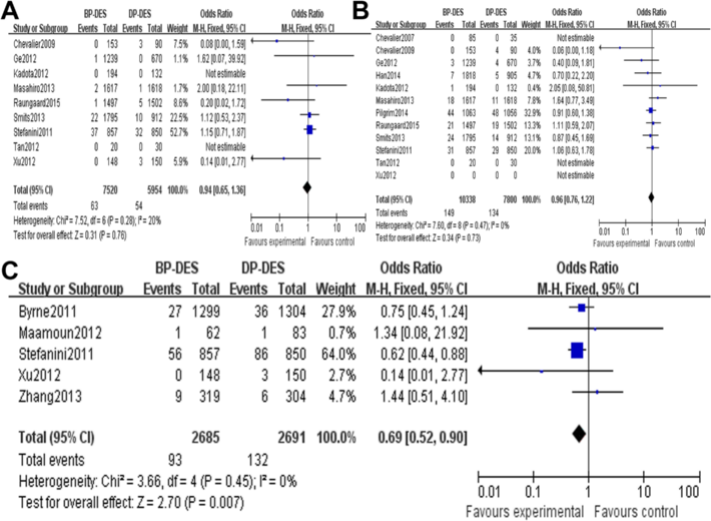
**Details regarding the different times of ST-related meta-analyses. (A)** The early ST forest plot for patients with coronary artery heart disease who received a BP-DES or a DP-DES. **(B)** The late ST forest plot for patients with coronary artery heart disease who received a BP-DES or a DP-DES. **(C)** The very late ST forest plot for patients with coronary artery heart disease who received a BP-DES or a DP-DES.

There was no significant heterogeneity regarding total mortality among the included studies that addressed total mortality, cardiac deaths, MI events, occurrence of ST, and different times ST (*P* = 0.84, *I*^2^ = 0%; *P* = 0.97, *I*^2^ = 0%; *P* = 1.00, *I*^2^ = 0%; *P* = 0.47, *I*^2^ = 0%; *P* = 0.28, *I*^2^ = 20%; *P* = 0.56, *I*^2^ = 0%; *P* = 0.45, *I*^2^ = 0%); therefore, a fixed effects model was used for all these incidences. There was no significant differences between the BP-DES and DP-DES groups with respect to overall mortality (OR 0.93, 95% CI 0.79 to 1.09, *Z* = 0.92, *P* = 0.36, see Figure 2C), cardiac deaths (OR 0.99, 95% CI 0.80 to 1.21, *Z* = 0.13, *P* = 0.90, Figure 5A), MI events and (OR 1.02, 95% CI 0.86 to 1.21, *Z* = 0.22, *P* = 0.82, Figure 5B), occurrence of ST (OR: 0.89, 95% CI: 0.72 to 1.09, Z = 1.16, *P* = 0.25, Figure 5C) during follow-up periods. However, very late ST (>12 months) was significantly lower in the BP-DES group than in the DP-DES group (OR 0.69, 95% CI 0.52 to 0. 90, *Z* = 2.70, *P* = 0.007). Details regarding the different times of ST-related meta-analyses are provided in Figure 6A, B, C.

### Publication bias analysis

**Figure 7:**
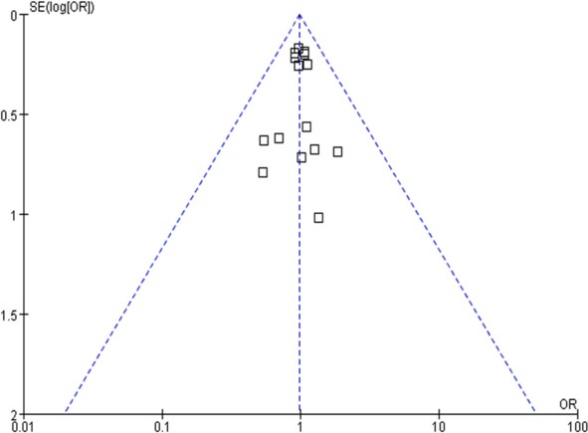
**A funnel plot indicating stent thrombosis among patients with coronary artery heart disease who received a BP-DES or a DP-DES.**

Figure 7 is the funnel plot indicating MI among patients with coronary artery heart disease who received a BP-DES or DP-DES. As indicated by the plot, no publication bias appeared to exist in the relevant studies.

We conducted meta-analyses of 16 RCTs; these studies examined a total of 22,211 patients, including 12,368 patients treated with BP-DESs and 9,843 patients treated with DP-DESs. Our study found no significant differences in cardiac death, overall mortality, MI, TLR, or TVR between patients treated with BP-DESs and patients treated with DP-DESs. However, the occurrence of very late ST (>12 months) may be significantly reduced by the use of BP-DESs instead of DP-DESs, and the general trend of the rates of TVR and TLR of DP-DESs groups was higher than BP-DESs groups after a 1-year follow-up. In addition, LLL were significantly less likely to occur in patients treated with BP-DESs than in patients treated with DP-DESs during follow-up periods. Our meta-analysis included abundant outcome indicators that can comprehensively reflect the efficacy of two different types of stents. It also addressed various types of BP-DESs, except for some specialized stent technologies (such as stents with reservoirs). As these stents are not strictly BP-DESs, a similar meta-analysis by Lupi *et al*. [37] excluded studies that addressed the use of specialized stent technologies (such as stents with reservoirs) from the original literature. Additionally, we included a few Chinese studies in our meta-analysis for detailed assessment and to reduce bias.

The results of this meta-analysis with respect to cardiac death, overall mortality, and MI are consistent with the findings from large-scale studies by Klauss *et al*. [38] and Byrne *et al*. [39] who indicated that there were no significant differences between BP-DESs and DP-DESs. However, the use of BP-DESs instead of DP-DESs can significantly reduce the incidence of both identified and potential cases of ST, as defined by the US and European Academic Research Consortium [40]. DES-induced delay of endothelialization is a factor that could increase the risk of thrombosis. The persistent stimulation of vascular endothelial cells by the permanent coating of DP-DESs can significantly delay the postoperative re-endothelialization of a target vessel after PCI, whereas the biological coating of BP-DESs completely degrades after the drug release process has completed, thus reducing the stimulation of these endothelial cells. A previous study has demonstrated that complete target lesion re-endothelialization occurs in 28 days after BMS implantation, but requires a significantly longer period of about 90 to 180 days after DES implantation [41]. The incidence of late ST is closely related to various factors, including early antiplatelet treatment, the extent of coronary artery lesions, and the quantity of implanted coronary stents [42]. However, long-term stimulation by permanent polymers is certainly an extremely important determinant of late ST.

Additionally, LLL during follow-up periods was significantly lower among patients treated with BP-DESs than among patients treated with DP-DESs. LLL is determined based on the difference between the minimum lumen diameter (MLD) immediately after stent implantation and the follow-up MLD after a follow-up period of at least 6 months.

To summarize, BP-DESs are safe and effective; additionally, they may become a valid alternative to DP-DESs. The incidence of ST increases after 1 year following the implantation of a DES. The follow-up periods of the studies examined in this investigation ranged from 6 to 48 months. To incorporate additional clinical studies with long follow-up times into meta-analyses of this topic and thereby obtain more stable and reliable conclusions, it is necessary to conduct additional large-scale rigorous RCTs with lengthy follow-up durations.

This meta-analysis exhibited the following limitations. (1) The included studies do not have identical follow-up periods; instead, the range of follow-up durations was relatively broad (between 6 and 48 months). (2) The limitations of the meta-analytical approach are well known and documented; thus, the safety and efficacy of various types of stents was not specifically identified [19].

## Conclusions

In conclusion, the meta-analysis results indicated that the implantation of BP-DESs can effectively prevent the incidence of mortality, cardiac death, MI, TLR, and other adverse events. The use of BP-DESs instead of DP-DESs can decrease the incidence of very late ST and alleviate LLL. In addition, the general trend of the rates of TVR and TLR of DP-DESs groups was higher than BP-DESs groups after a 1-year follow-up. To summarize, BP-DESs are safe and effective.

## Abbreviations

BP-DESs: biodegradable polymer drug-eluting stents
DP-DESs: durable polymer drug-eluting stents
TLR: target lesion revascularization
TVR: target vessel revascularization
LLL: late lumen loss
WMD: weighted mean difference
MI: myocardial infarction
ST: stent thrombosis
DESs: drug-eluting stents
BMSs: bare metal stents
PCI: percutaneous coronary intervention
ST: stent thrombosis
CIs: confidence intervals
MLD: minimum lumen diameter
